# Unilateral electrical stimulation of the heart 7 acupuncture point to prevent emergence agitation in children: A prospective, double-blinded, randomized clinical trial

**DOI:** 10.1371/journal.pone.0204533

**Published:** 2018-10-10

**Authors:** Nobuhito Nakamura, Takahiro Mihara, Toshiyuki Hijikata, Takahisa Goto, Koui Ka

**Affiliations:** 1 Department of Anesthesiology, Kanagawa Children’s Medical Center, Yokohama, Japan; 2 Department of Anesthesiology and Critical Care Medicine, Yokohama City University Graduate School of Medicine, Yokohama, Japan; University of Tokyo, JAPAN

## Abstract

**Background:**

Emergence agitation (EA) is a frequent phenomenon in children recovering from general anaesthesia and increases the risk of self-injury. Previously, our group reported that stimulating the heart 7 (HT7) acupuncture point bilaterally using two neuromuscular transmission monitoring devices (NTMs) decreased the incidence of EA. However, bilateral stimulation is a barrier to clinical use because two NTMs are needed for one patient.

**Objective:**

The objective of this study was to examine the efficacy of unilateral electrical stimulation of HT7 using an NTM to prevent EA in children.

**Design:**

Prospective, double-blinded, randomized clinical trial.

**Setting:**

Kanagawa Children’s Medical Centre, Yokohama, Japan.

**Patients:**

One hundred children (ages 18–96 months) with ASA-PS I or II, who were scheduled to undergo inguinal hernia repair or orchiopexy under sevoflurane anaesthesia.

**Intervention:**

Patients were randomly assigned to one of the following two groups: (1) HT7 group: unilateral (right side) stimulation of the HT7 acupuncture point using a single-twitch electrical stimulus (1 Hz, 50 mA) throughout the surgery, and (2) control group: electrodes alone were attached to the HT7 point on the right side; an electrical stimulus was not applied.

**Main outcome measures:**

The primary outcome was the incidence of EA evaluated using the pediatric anaesthesia emergence delirium (PAED) scale. The secondary outcomes were the incidence of EA evaluated using Aono’s scale, the severity of EA, PACU stay duration, and postoperative pain.

**Results:**

There was no statistical difference between the incidence of EA in the HT7 and the control group (28.0% and 24.0%, respectively; P > 0.99). The risk ratio was 1.17 (95% confidence interval: 0.60–2.27).

**Conclusions:**

We observed that there was no effect of unilateral single-twitch electrical stimulation to the HT7 on the incidence of EA, contrary to the findings with bilateral HT7 stimulation.

## Introduction

Emergence agitation (EA) is a major postoperative problem in pediatric anaesthesia[1]. Prevention of EA is important because patients may injure themselves by hitting against the bed rails or removing their catheter or drainage tube. Moreover, EA increases the risk of postoperative maladaptive behavior[2]. The mainstay of prevention of EA is drug therapy, such as propofol or fentanyl[3–5]. However, these drugs have side effects such as delayed recovery and respiratory depression[5,6]. Therefore, alternative methods for preventing EA are preferred.

Acupuncture is considered to be one solution without pharmacological therapy.

Acupuncture at the heart 7 (HT7) which is located on the ulnar side of the wrist seems to be effective in treating anxiety[7]. This may aid in preventing EA because preoperative anxiety is correlated with the incidence of EA. Actually, HT7 point stimulation has been reported to decrease EA. Previous studies have reported that stimulating HT7 acupuncture point using a capsicum plaster[8] or a needle[9] prevented EA in children. In addition, our group found that stimulating the HT7 acupuncture point bilaterally using a neuromuscular transmission monitor decreased the incidence of EA[10].

Acustimulation using a neuromuscular transmission monitor may be easy to perform because many anesthesiologists are familiar with this device compared to using a capsicum plaster or needle. However, in the previous study, bilateral HT7 stimulation was performed, which is a barrier to clinical use because two machines are needed for one patient. To utilize this method in a clinical setting, the conditions by which HT7 is electrically stimulated must be optimized.

The objective of this study was to examine the efficacy of unilateral electrical stimulation of HT7 using a neuromuscular transmission monitor (NTM) to prevent EA in pediatric patients.

## Materials and methods

Approval was obtained by the institutional review board of Kanagawa Children’s Medical Centre (No. 1308-07) on February 25, 2014. The study protocol was registered in the UMIN Clinical Trial Registry (registration number UMIN000014729, principal investigator N. Nakamura, date of registration July 31, 2014). Written informed consent was obtained from the parents of all patients. The study was conducted in accordance with the principles of the Declaration of Helsinki. This study took place at Kanagawa Children`s Medical Center from October 2014 to August 2015.

This study was a prospective, double-blinded, parallel-group, randomized clinical trial. We recruited patients who were scheduled to undergo inguinal hernia repair or orchiopexy under sevoflurane anesthesia. Children were 18–96 months of age, with American Society of Anesthesiologists physical status I or II. Exclusion criteria were developmental delay, neurological or psychological disorders, abnormal airways, reactive airway diseases and usage of sedatives. We assume that there is no laterality on the effect of unilateral stimulation of HT7. We choose right hand to stimulate HT7 in this study. The enrolled patients were randomly assigned to one of the following two groups: in the HT7 group, unilateral right-side stimulation of the HT 7 acupuncture point was performed on patients using a single-twitch electrical stimulus throughout the surgery; in the control group, electrodes were attached to HT7 point on the right side, and an electrical stimulus was not applied.

Group assignment was determined by opening a sealed, opaque, sequentially numbered envelope with a computer-generated random allocation. Both patients and observers were blinded to group allocation. Observers did not participate in anesthetic care in the operating room. Attending anesthesiologists opened the envelope after the completion of anesthesia induction. Attending anesthesiologists could not be blinded after starting electrical stimulation of HT7 because we don’t use the neuromuscular blockade in this trial. Electrical stimulation of the HT7 evoked movements of the patients’ hand, whereas patients in the control group did not move. To reduce bias, we used a standardized anesthetic protocol throughout the trial.

Patients were not premedicated. We assessed patient’s anxiety using the short version of the modified Yale Preoperative Anxiety Scale (m-YPAS)[11,12] before induction of anesthesia. Standard monitoring devices such as pulse oximetry, electrocardiography, and a blood pressure cuff were attached to patients before induction. Anesthesia was induced by sevoflurane in 4 l/min nitrous oxide and 2 l/min oxygen. Sevoflurane concentration was gradually increased to a maximum of 8%. After loss of consciousness, an intravenous (I.V.) line was inserted. Atropine (0.01 mg/kg) and fentanyl (1 μg/kg) were administrated via the I.V. line. A laryngeal mask airway (LMA-Proseal; Teleflex Medical; Research Triangle Park, NC) or tracheal tube was inserted, and sevoflurane was adjusted to end-tidal concentration of 2–3%. The laryngeal mask airway (LMA) size was determined according to the manufacturer’s guidelines. After insertion of the LMA or tracheal intubation, caudal blocks with 1 ml/kg (up to a maximum of 20 mL) of 0.25% ropivacaine or an ilioinguinal block with 0.3 mL/kg of a 1:1 mixture of 0.375% ropivacaine and 0.5% lidocaine, were performed. Anesthesia was maintained with 2–3% of sevoflurane in 1 l/min of oxygen and 3 l/min of air.

During the operation, if the heart rate or blood pressure increased by more than 10% of the baseline data, 1 μg/kg of fentanyl was administered. After the operation was completed, 100% oxygen was administered and the tracheal tube or LMA was removed under deep anesthesia when adequate spontaneous ventilation was confirmed (i.e., tidal volume of more than 5 ml/kg). After removal of the tracheal tube or LMA, the attending anesthesiologist confirmed that the patient was breathing spontaneously without any respiratory complications such as holding their breath, upper airway obstruction, or laryngospasm. The patients were transferred to the post-anesthesia care unit (PACU) with a stable airway and appropriate ventilation.

The HT7 acupuncture point is located at the wrist crease on the radial side of the flexor carpi ulnaris tendon, between the ulna and pisiform bones. After the induction of anesthesia, electrodes were attached to the patient on the right arm, one electrode on the HT7 acupuncture point and another on the dorsal side of the acupuncture point (S1 Fig). In the HT7 stimulation group, single-twitch stimulation at 1 Hz (0.2 ms, 50 mA) was applied to the HT7 acupuncture site during the surgery using an NTM device (TOF-Watch; Mammendorfer Institute of Physics and Medicine, Mammendorf, Germany). We connected the negative cable of the NTM device to the electrode on the HT7 acupuncture point and the positive cable to the electrode on the dorsal side. We intended electrical current to flow through the HT7 acupuncture point. In the control group, an NTM device was similarly connected to the electrode, but electrical stimulation was not applied.

The primary outcome was the incidence of EA, evaluated by the Pediatric Anesthesia Emergence Delirium (PAED) scale[13]. The secondary outcomes were the incidence of EA evaluated using Aono’s scale[14], the severity of EA evaluated by PAED scale[13] or Aono’s scale[14], PACU stay duration, and postoperative pain.

Trained observers, who were blinded to the patient allocations, assessed and recorded the patient’s recovery condition, including the PAED score in the PACU. Patients were evaluated every 5 min from the time they opened their eyes in response to a verbal command or light touch, until they left the PACU. The theoretical scores on the PAED scale range from 0 to 20; on Aono’s scale, the scores range from 1 to 4. If the highest recorded score while in the PACU was ≥ 10 on the PAED scale or ≥ 3 on Aono’s scale, this was considered as EA[13, 14]. The observers evaluated postoperative pain using the Children’s Hospital of Eastern Ontario Pain Scale (CHEOPS). Discharge from PACU were allowed when the patient’s modified Aldrete score was more than 8 points[15, 16]. Adverse effects of acupuncture site stimulation (pain, swelling, redness or skin rush), duration of PACU stay, duration of operation, and duration of anesthesia (from the start of induction to the end of administration of oxygen using a closed-circuit anesthesia machine) were also recorded.

### Statistical analysis

We used the data from the previous study by our group[10] for sample size calculation. The incidence of EA in patients who underwent inguinal hernia repair or orchiopexy was 15.4% in the bilateral HT7 stimulation group and 43.6% in the control group, respectively. We hypothesized that unilateral stimulation has a similar effect as bilateral stimulation. A power analysis (α = 0.05, β = 0.20) indicated that 46 patients were required in each group. We recruited 50 patients into each group, considering a 10% dropout rate.

Statistical analyses were performed using the R statistical software package, version 3.0.2 (R Foundation for Statistical Computing, Vienna, Austria).

The Shapiro–Wilk test was used to assess the normality of the data distributions.

Data distributed consistent with normality are reported as mean ± standard deviation and were analyzed using unpaired *t*-tests. Categorical data are reported as percentages, and were analyzed using Fisher’s exact test or the chi-squared test as appropriate. Data not distributed consistent with normality are reported as the median and interquartile range (IQR), and were analyzed using the Mann–Whitney *U* test. The risk ratio (RR) with a 95% confidence interval (CI) was calculated for our primary outcome (i.e. the incidence of EA as evaluated using the PAED scale). Statistical significance was declared at the 0.05 level.

## Results

**Fig 1:**
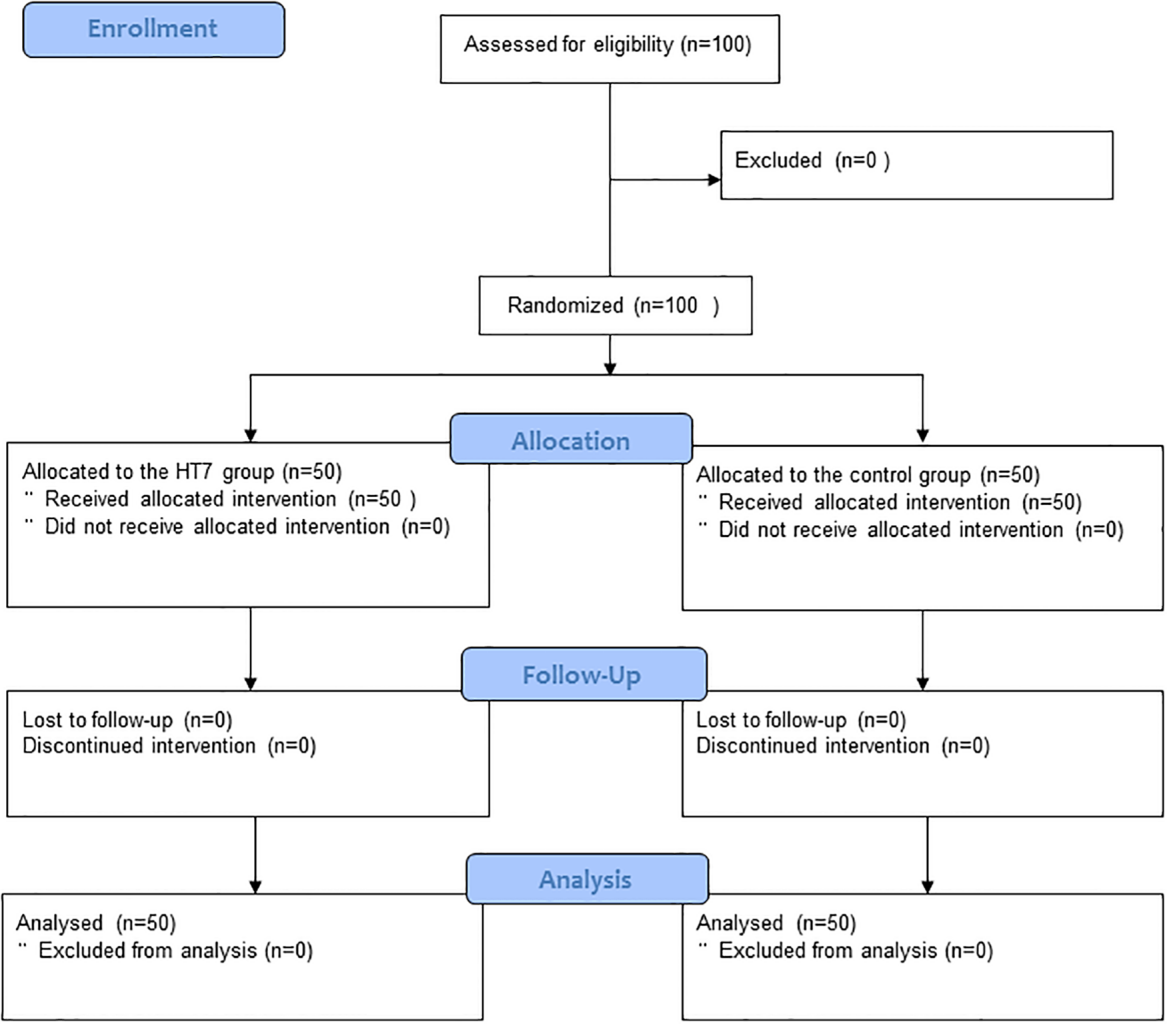
A CONSORT patient flow diagram.

All 100 enrolled subjects completed the study (Fig 1) and recruitment of patients was stopped because we reached the target sample size (i.e., 100 patients). Patient characteristics, operation type, short version of the m-YPAS before anesthesia induction, operation duration, anesthesia duration, and fentanyl consumption were comparable between the two groups (Table 1).

**Table 1:** Patients characteristics.

	HT7^a^ (*n* = 50)	Control (*n* = 50)	*P* value
Age (months)	45 (27–60)	52 (31–74)	0.13
Sex (male:female)	37:13	37:13	> 0.99
Weight (kg)	14.3 (12.6–17.6)	16.2 (12.7–19.5)	0.27
Type of operation			0.11
inguinal hernia repair	33	41	
other minor surgery^b^	17	9	
Airway management			>0.99
LMA	47	46	
Tracheal intubation	3	4	
Preoperative behaviour	2 (1–3)	2 (1–3)	0.38
Operation time (min)	24 (15–67)	23 (14–45)	0.20
Anaesthesia time (min)	55 (40–119)	51 (40–87)	0.32
Consumption of fentanyl (μg)	20 (15–24)	15 (15–24)	0.24

Data are the median (interquartile range) or number

^a^HT7: heart 7 acupuncture point

^**b**^other minor surgery: 24 cases of orchiopexy, 1 case of hydrocelectomy, and 1 case of umbilical hernia repair.

There was no statistical difference between the incidence of EA using the PAED scale in the HT7 and the control groups (28.0% and 24.0%, respectively; P > 0.99). The risk ratio (95% CI) was 1.17 (0.60–2.27) (Table 2). A similar result was obtained using Aono’s scale (28.0% and 26.0%, respectively; *P* > 0.99) (Table 3).

**Table 2:** Incidence of EA assessed by PAED scale^c^.

	HT7^d^ (n = 50) % (n)	Control (n = 50) % (n)	RR^a^ [95% CI]	*P* value
EA^b^ present	28.0 (14/50)	24.0 (12/50)	1.17 [0.60–2.27]	> 0.99

^a^RR: risk ratio

^b^EA: emergence agitation

^c^PAED: pediatric anesthesia emergence delirium scale

^d^HT7: heart 7 acupuncture point.

Values are proportion (number of patients with EA/ total number) or point estimate [95% confidence interval].

**Table 3:** Results of secondary outcomes.

	HT7^a^ (*n* = 50) % (n) or median [IQR]	Control (*n* = 50) % (n) or median [IQR^b^]	*P* value
Incidence of EA^c^ (Aono’s ≥ 3)	28.0 (14)	26.0 (13)	> 0.99
Aono’s score	1 [1–3]	1 [1–3]	0.33
PAED^d^ score	4 [0–11.5]	0.5 [0–8.5]	0.14
PACU^e^ stay duration (min)	35 [26–43]	37 [29–46]	0.28
CHEOPS^f^ score	7 [6–11]	7 [5–9]	0.30

^a^HT7: heart 7 acupuncture point

^b^IQR: interquartile range

^c^EA: emergence agitation

^d^PAED: pediatric anesthesia emergence delirium scale

^e^PACU: post-anesthesia care unit

^f^CHEOPS: Children’s Hospital of Eastern Ontario Pain Scale.

There was no statistically significant difference between the PAED scores between the HT7 and control groups (the median [IQR] scores were 4 [0–11.5] and 0.5 [0–8.5] in the HT7 and control group, respectively; P = 0.14) (Table 3). Duration of stay in the PACU was comparable between the two groups. The CHEOPS scores did not differ significantly between the two groups (7 [6–11] and 7 [5–9] in the HT7 and control group, respectively; P = 0.30) (Table 3). No adverse effects of stimulation such as pain, paralysis, or redness occurred during the study.

## Discussion

In this study, we showed that unilateral electrical stimulation of the HT7 did not have a significant effect on preventing EA. The severity of EA was similar to the control group, in which no electrical stimulation was applied. No adverse event was produced by electrical stimulation.

Although we could not confirm the effect of unilateral HT7 stimulation on preventing EA, several studies have reported the effect of bilateral HT7 stimulation to prevent EA[8–10]. The reason for the discrepancy of the effect between the unilateral and bilateral HT7 stimulation is unclear. There are limited studies reporting the effect of HT7 and its role in preventing EA. Knowledge regarding this type of acupuncture is currently lacking. We speculate that the mode and amount of stimulation play an important role in the effect. A previous study[17] revealed that the effect of unilateral stimulation of the P6 acupoint using NTM to prevent postoperative nausea and vomiting was greater in the tetanic stimulation group as compared to other groups with different modes of stimulation, including the single twitch group. The results of P6 stimulation clearly indicated that the effect of acustimulation depends on the mode and amount of stimulation. In the current study, we applied single-twitch stimulation (1 Hz, 50 mA) to the unilateral HT7 acupoint. This method may not be suitable for unilateral stimulation. Further studies should be performed to investigate whether other modes of stimulation, such as tetanus stimulation, are effective in the prevention of EA.

In this study, we applied stimulation to the HT7 under general anesthesia because children would not tolerate electrical stimulation while awake. However, stimulation under general anesthesia may have diminished the effect of acustimulation[18, 19]. We consider that acupressure using a pressure band[20, 21] would be possible for children who are conscious. Because adverse events such as delayed recovery or respiratory depression were not observed following HT7 stimulation, we believe that further studies investigating the optimal method of HT7 stimulation for preventing EA should be performed.

This study has several strengths. First, we evaluated EA using both the PAED scale and Aono’s scale. Although the PAED scale is the only validated scale to measure EA[13], a previous study showed that the incidence of EA was different depending on the tools used for evaluation[22]. Therefore, we used not only the PAED scale but also Aono’s scale. In this study, there was no difference in the incidence of EA between the HT7 and control groups regardless of the scale used for evaluating EA. Second, we performed a caudal or ilioinguinal nerve block for all participants. As postoperative pain is one of the confounding factors of EA[23, 24], it is necessary to control pain to eliminate confounding. In this protocol, we performed nerve blocks in all patients, and thus, there was little need for an additional intraoperative opioid that may have had an effect on EA. Consequently, there were no differences in the consumption of fentanyl during the operation, or in the CHEOPS score between the two groups.

This study has some limitations. First, the attending anesthesiologists could not be blinded to the group allocations after starting electrical stimulation of HT7. As we did not use a neuromuscular blocking agent, hand motion in patients provoked by electrical stimulation of the HT7 revealed the group allocations. Therefore, we used a standard anesthetic protocol to reduce bias in this study. In addition, to ensure data integrity, the patients and the observers were blinded to the group allocations. Second, we enrolled patients who were scheduled to undergo hernia repair or orchiopexy under sevoflurane anesthesia. We cannot extrapolate the results of this study to patients who received propofol anesthesia or those undergoing other operative procedures. Third, the current study may be underpowered because power analysis was performed based on the data of previous study on our group. In this study, incidence of EA in control group was lower than previous study of our group (24% and 43.6%, respectively). This could be another reason for having obtained negative results in this study.

## Conclusion

In conclusion, there was no difference in the incidence of EA between the unilateral HT7 stimulation group and the control group, contrary to previous findings with bilateral HT7 stimulation. Further research is warranted to elucidate all the effects of HT7 stimulation.

## Supporting information

S1 Fig Stimulation of the heart 7 acupuncture point (HT7) using neuromuscular transmission monitor (NTM). A: One NTM electrode was attached just above the HT7 acupuncture point. B: Another electrode was attached on the dorsal side of the HT7 acupuncture point. We stimulated HT7 on both sides using NTM (TOF-Watch). Single-twitch stimulation at 1 Hz (over 0.2 ms, at a constant current of 50 mA) was applied throughout the operation.
(TIF)

S1 Table Data of the patients included in this study. (DOCX)

S1 File CONSORT 2010 checklist. (DOC)

S2 File Protocol (English version). (DOCX)

S3 File Original protocol in Japanese. (DOCX)
