# Supplementary figures and images for: Unilateral electrical stimulation of the heart 7 acupuncture point to prevent emergence agitation in children: A prospective, double-blinded, randomized clinical trial
Source: PLoS One. 2018 Oct 10;13(10):e0204533. doi: 10.1371/journal.pone.0204533 (PMC6179240; doi:10.1371/journal.pone.0204533)

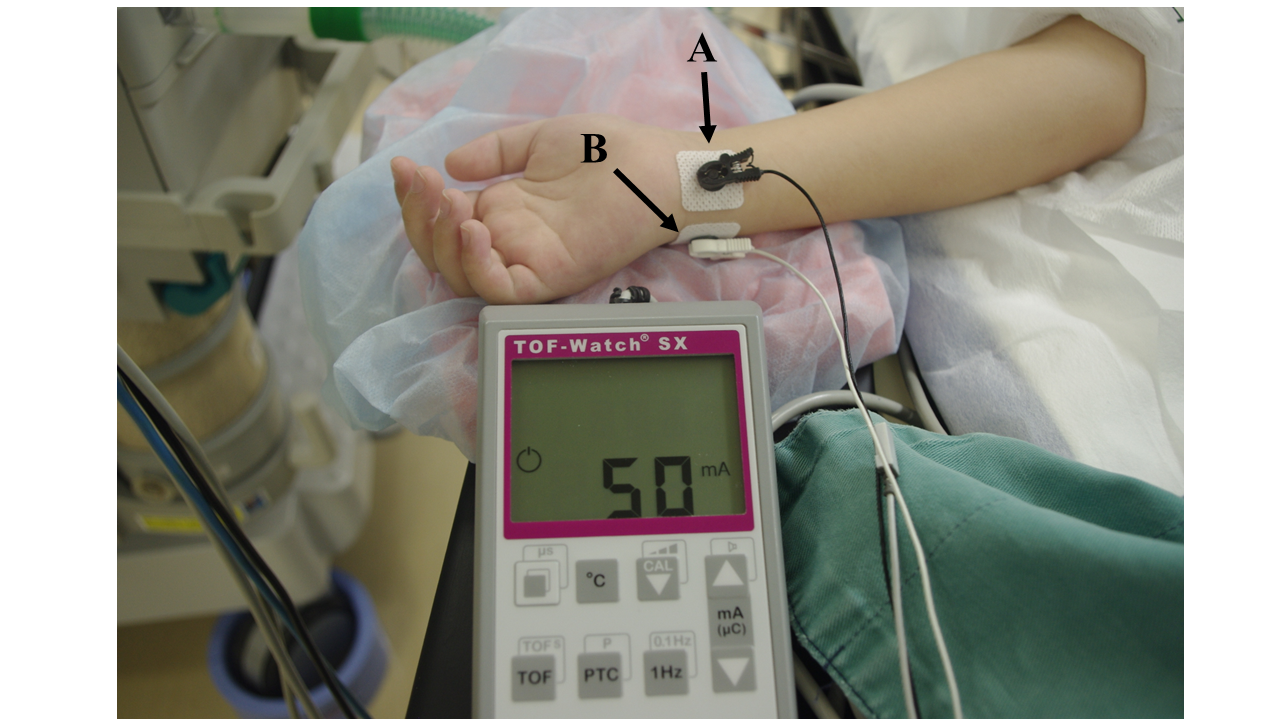

Supplement: S1 Fig — A: One NTM electrode was attached just above the HT7 acupuncture point. B: Another electrode was attached on the dorsal side of the HT7 acupuncture point. We stimulated HT7 on both sides using NTM (TOF-Watch). Single-twitch stimulation at 1 Hz (over 0.2 ms, at a constant current of 50 mA) was applied throughout the operation. (TIF) [file pone.0204533.s001.tif]
