# Supplementary material for: Unilateral electrical stimulation of the heart 7 acupuncture point to prevent emergence agitation in children: A prospective, double-blinded, randomized clinical trial
Source: PLoS One. 2018 Oct 10;13(10):e0204533. doi: 10.1371/journal.pone.0204533 (PMC6179240; doi:10.1371/journal.pone.0204533)
Supplement: S1 Table — (DOCX) [file pone.0204533.s002.docx]

S1 Table. Data of the patients included in this study.

Data were arranged in the following order from left; (1) Group; (2) Sex; (3) Surgical procedure; (4) Analgetic Blocks; ( IIB: Ilioinguinal block, RSB: Rectus sheeth block); (5) Weight; (6) Consumption of fentanyl; (7) Anesthesia time; (8) Operation time; (9) Preoperative behaviour; (10) PAED (pediatric anesthesia emergence delirium scale) score; (11) Emergence agitation assessed by PAED scale; (12) Aono’s score; (13) Emergence agitation assessed by Aono’s scale; (14) Children’s Hospital of Eastern Ontario Pain Scale; (15) Recovery time;

| HT7 | male | orchiopexy | caudal | 12 | 10 | 176 | 125 | 2 | 4 | 0 | 2 | 0 | 7 | 27 |
| --- | --- | --- | --- | --- | --- | --- | --- | --- | --- | --- | --- | --- | --- | --- |
| Control | male | inguinal hernia repair | IIB | 11 | 10 | 63 | 26 | 2 | 5 | 0 | 2 | 0 | 8 | 27 |
| Control | male | orchiopexy | caudal | 9.8 | 10 | 104 | 66 | 3 | 16 | 1 | 4 | 1 | 11 | 34 |
| HT7 | female | inguinal hernia repair | IIB | 15.7 | 15 | 40 | 14 | 2 | 4 | 0 | 2 | 0 | 7 | 46 |
| HT7 | male | inguinal hernia repair | IIB | 14.7 | 15 | 43 | 18 | 2 | 14 | 1 | 3 | 1 | 12 | 28 |
| Control | male | inguinal hernia repair | IIB | 14.1 | 15 | 42 | 12 | 1 | 0 | 0 | 1 | 0 | 5 | 59 |
| HT7 | male | inguinal hernia repair | IIB | 9.7 | 20 | 43 | 23 | 3 | 8 | 0 | 1 | 0 | 9 | 53 |
| Control | female | inguinal hernia repair | IIB | 21 | 20 | 40 | 18 | 1 | 0 | 0 | 1 | 0 | 5 | 40 |
| Control | male | orchiopexy | caudal | 19 | 20 | 92 | 40 | 2 | 2 | 0 | 1 | 0 | 7 | 36 |
| HT7 | male | orchiopexy | caudal | 16 | 15 | 75 | 32 | 2 | 14 | 1 | 3 | 1 | 11 | 24 |
| Control | male | inguinal hernia repair | IIB | 17.5 | 15 | 49 | 19 | 1 | 0 | 0 | 1 | 0 | 6 | 49 |
| HT7 | male | inguinal hernia repair | IIB | 14.3 | 20 | 39 | 14 | 1 | 3 | 0 | 2 | 0 | 12 | 36 |
| HT7 | male | inguinal hernia repair | IIB | 16.4 | 15 | 37 | 16 | 1 | 0 | 0 | 1 | 0 | 5 | 36 |
| Control | male | inguinal hernia repair | IIB | 23.5 | 25 | 40 | 16 | 1 | 0 | 0 | 1 | 0 | 6 | 41 |
| Control | female | inguinal hernia repair | IIB | 12.6 | 15 | 31 | 10 | 2 | 0 | 0 | 1 | 0 | 5 | 53 |
| HT7 | female | inguinal hernia repair | IIB | 15.6 | 15 | 47 | 27 | 3 | 0 | 0 | 1 | 0 | 5 | 21 |
| Control | male | inguinal hernia repair | IIB | 17.6 | 20 | 32 | 13 | 1 | 14 | 1 | 3 | 1 | 12 | 21 |
| HT7 | male | inguinal hernia repair | IIB | 13 | 15 | 35 | 16 | 3 | 12 | 1 | 3 | 1 | 11 | 38 |
| Control | male | inguinal hernia repair | IIB | 15.8 | 45 | 50 | 27 | 3 | 3 | 0 | 1 | 0 | 5 | 46 |
| Control | male | orchiopexy | caudal | 8.9 | 10 | 43 | 14 | 3 | 14 | 1 | 3 | 1 | 10 | 29 |
| HT7 | male | orchiopexy | caudal | 9.9 | 10 | 123 | 76 | 1 | 12 | 1 | 4 | 1 | 10 | 27 |
| HT7 | male | inguinal hernia repair | IIB | 10.9 | 10 | 42 | 19 | 3 | 10 | 1 | 3 | 1 | 11 | 33 |
| HT7 | female | inguinal hernia repair | IIB | 19.6 | 20 | 37 | 12 | 1 | 5 | 0 | 1 | 0 | 5 | 24 |
| HT7 | male | orchiopexy | caudal | 13.6 | 15 | 163 | 115 | 3 | 3 | 0 | 2 | 0 | 10 | 28 |
| HT7 | female | inguinal hernia repair | IIB | 14.3 | 15 | 32 | 10 | 2 | 0 | 0 | 1 | 0 | 7 | 36 |
| Control | female | inguinal hernia repair | IIB | 14.8 | 15 | 60 | 35 | 3 | 0 | 0 | 1 | 0 | 6 | 52 |
| Control | female | inguinal hernia repair | IIB | 17.4 | 15 | 31 | 10 | 1 | 0 | 0 | 1 | 0 | 6 | 29 |
| Control | male | inguinal hernia repair | IIB | 14 | 15 | 48 | 22 | 3 | 7 | 0 | 2 | 0 | 7 | 36 |
| HT7 | male | inguinal hernia repair | IIB | 24.8 | 25 | 124 | 19 | 1 | 0 | 0 | 1 | 0 | 5 | 26 |
| Control | female | inguinal hernia repair | IIB | 18.7 | 20 | 35 | 13 | 1 | 0 | 0 | 1 | 0 | 5 | 26 |
| HT7 | male | inguinal hernia repair | IIB | 27.6 | 25 | 35 | 12 | 1 | 0 | 0 | 1 | 0 | 5 | 38 |
| HT7 | male | inguinal hernia repair | IIB | 13.9 | 15 | 34 | 11 | 3 | 0 | 0 | 1 | 0 | 7 | 24 |
| Control | male | inguinal hernia repair | IIB | 20.9 | 20 | 36 | 14 | 1 | 15 | 1 | 3 | 1 | 9 | 25 |
| Control | male | inguinal hernia repair | IIB | 24.7 | 25 | 46 | 20 | 1 | 2 | 0 | 1 | 0 | 9 | 46 |
| HT7 | male | inguinal hernia repair | IIB | 20 | 50 | 88 | 52 | 1 | 3 | 0 | 1 | 0 | 10 | 64 |
| Control | male | inguinal hernia repair | IIB | 11 | 10 | 52 | 23 | 3 | 0 | 0 | 1 | 0 | 6 | 82 |
| Control | male | inguinal hernia repair | IIB | 10.6 | 10 | 75 | 47 | 2 | 12 | 1 | 3 | 1 | 9 | 37 |
| Control | male | inguinal hernia repair | IIB | 16.6 | 15 | 44 | 15 | 1 | 0 | 0 | 1 | 0 | 6 | 36 |
| Control | female | inguinal hernia repair | IIB | 19.6 | 20 | 33 | 12 | 1 | 3 | 0 | 1 | 0 | 6 | 34 |
| HT7 | male | orchiopexy | caudal | 16 | 15 | 110 | 65 | 1 | 0 | 0 | 1 | 0 | 7 | 29 |
| HT7 | male | inguinal hernia repair | IIB | 12 | 30 | 47 | 19 | 3 | 16 | 1 | 4 | 1 | 13 | 19 |
| HT7 | male | orchiopexy | caudal | 14 | 50 | 173 | 120 | 3 | 2 | 0 | 1 | 0 | 6 | 60 |
| Control | female | inguinal hernia repair | IIB | 14.9 | 15 | 47 | 11 | 3 | 20 | 1 | 4 | 1 | 12 | 20 |
| HT7 | male | inguinal hernia repair | IIB | 17.3 | 30 | 38 | 18 | 3 | 8 | 0 | 3 | 1 | 10 | 43 |
| Control | male | orchiopexy | caudal | 12.9 | 10 | 113 | 68 | 2 | 20 | 1 | 3 | 1 | 11 | 23 |
| Control | male | inguinal hernia repair | IIB | 14.5 | 15 | 91 | 70 | 3 | 4 | 0 | 1 | 0 | 7 | 49 |
| HT7 | male | inguinal hernia repair | IIB | 21.9 | 20 | 42 | 15 | 1 | 0 | 0 | 1 | 0 | 7 | 38 |
| HT7 | male | orchiopexy | caudal | 15.5 | 70 | 241 | 174 | 2 | 19 | 1 | 4 | 1 | 7 | 19 |
| Control | male | orchiopexy | caudal | 12.9 | 15 | 112 | 65 | 3 | 20 | 1 | 4 | 1 | 12 | 24 |
| Control | male | inguinal hernia repair | IIB | 12 | 10 | 50 | 25 | 2 | 7 | 0 | 2 | 0 | 7 | 33 |
| Control | male | orchiopexy | caudal | 19.9 | 40 | 140 | 97 | 1 | 0 | 0 | 1 | 0 | 5 | 41 |
| HT7 | male | inguinal hernia repair | IIB | 28.2 | 25 | 82 | 46 | 1 | 20 | 1 | 4 | 1 | 13 | 33 |
| Control | male | inguinal hernia repair | IIB | 9.9 | 10 | 51 | 24 | 3 | 14 | 1 | 3 | 1 | 11 | 40 |
| Control | female | inguinal hernia repair | IIB | 19.2 | 20 | 39 | 14 | 1 | 0 | 0 | 1 | 0 | 4 | 31 |
| Control | female | inguinal hernia repair | IIB | 14.3 | 15 | 33 | 10 | 1 | 0 | 0 | 1 | 0 | 6 | 37 |
| Control | male | inguinal hernia repair | IIB | 21 | 40 | 63 | 33 | 1 | 0 | 0 | 1 | 0 | 5 | 34 |
| Control | male | inguinal hernia repair | IIB | 22 | 25 | 92 | 53 | 1 | 0 | 0 | 1 | 0 | 8 | 42 |
| HT7 | male | orchiopexy | IIB | 15.6 | 20 | 143 | 100 | 1 | 16 | 1 | 3 | 1 | 10 | 28 |
| Control | male | inguinal hernia repair | IIB | 18 | 15 | 62 | 35 | 1 | 0 | 0 | 1 | 0 | 6 | 24 |
| Control | male | inguinal hernia repair | IIB | 23.1 | 25 | 36 | 12 | 2 | 0 | 0 | 1 | 0 | 6 | 33 |
| HT7 | male | orchiopexy | caudal | 9.2 | 10 | 202 | 143 | 3 | 14 | 1 | 3 | 1 | 11 | 19 |
| Control | male | inguinal hernia repair | IIB | 10.6 | 20 | 52 | 23 | 3 | 12 | 1 | 3 | 1 | 8 | 30 |
| HT7 | male | orchiopexy | caudal | 12 | 15 | 231 | 171 | 3 | 5 | 0 | 2 | 0 | 12 | 29 |
| HT7 | male | orchiopexy | caudal | 12.4 | 20 | 102 | 60 | 2 | 7 | 0 | 1 | 0 | 6 | 64 |
| HT7 | female | inguinal hernia repair | IIB | 14.8 | 15 | 35 | 12 | 2 | 1 | 0 | 1 | 0 | 5 | 26 |
| HT7 | female | inguinal hernia repair | IIB | 15.4 | 20 | 29 | 9 | 1 | 2 | 0 | 1 | 0 | 5 | 35 |
| HT7 | female | inguinal hernia repair | IIB | 13.7 | 15 | 38 | 15 | 1 | 1 | 0 | 1 | 0 | 6 | 36 |
| HT7 | male | orchiopexy | caudal | 10.5 | 10 | 161 | 114 | 3 | 9 | 0 | 2 | 0 | 11 | 25 |
| HT7 | female | inguinal hernia repair | IIB | 21 | 20 | 40 | 15 | 1 | 0 | 0 | 1 | 0 | 5 | 40 |
| HT7 | male | orchiopexy | caudal | 13.3 | 15 | 81 | 36 | 1 | 6 | 0 | 2 | 0 | 11 | 41 |
| Control | female | inguinal hernia repair | IIB | 9.3 | 10 | 39 | 11 | 2 | 5 | 0 | 3 | 1 | 11 | 26 |
| Control | female | inguinal hernia repair | IIB | 14.1 | 15 | 42 | 18 | 1 | 0 | 0 | 1 | 0 | 6 | 27 |
| Control | male | inguinal hernia repair | IIB | 18.8 | 60 | 134 | 86 | 1 | 3 | 0 | 1 | 0 | 6 | 44 |
| Control | male | inguinal hernia repair | caudal | 23.3 | 25 | 97 | 48 | 2 | 0 | 0 | 1 | 0 | 9 | 34 |
| Control | male | orchiopexy | caudal | 12.5 | 15 | 180 | 125 | 3 | 0 | 0 | 2 | 0 | 9 | 45 |
| HT7 | female | inguinal hernia repair | IIB | 19.9 | 20 | 59 | 25 | 1 | 2 | 0 | 1 | 0 | 6 | 40 |
| HT7 | male | inguinal hernia repair | caudal | 9 | 20 | 121 | 75 | 3 | 4 | 0 | 2 | 0 | 7 | 27 |
| Control | male | inguinal hernia repair | IIB | 12.9 | 15 | 53 | 23 | 1 | 0 | 0 | 2 | 0 | 7 | 47 |
| Control | male | inguinal hernia repair | IIB | 22.8 | 25 | 55 | 26 | 1 | 0 | 0 | 1 | 0 | 8 | 30 |
| HT7 | male | inguinal hernia repair | IIB | 17.7 | 20 | 45 | 18 | 1 | 0 | 0 | 1 | 0 | 6 | 51 |
| HT7 | male | inguinal hernia repair | IIB | 13.7 | 30 | 52 | 22 | 1 | 1 | 0 | 1 | 0 | 7 | 72 |
| Control | male | inguinal hernia repair | caudal | 15 | 15 | 99 | 52 | 2 | 13 | 1 | 3 | 1 | 11 | 55 |
| Control | male | inguinal hernia repair | IIB | 11.4 | 10 | 41 | 15 | 2 | 0 | 0 | 1 | 0 | 5 | 22 |
| HT7 | male | inguinal hernia repair | IIB | 13.8 | 15 | 58 | 30 | 3 | 16 | 1 | 4 | 1 | 12 | 23 |
| HT7 | female | inguinal hernia repair | IIB | 20 | 40 | 35 | 13 | 2 | 0 | 0 | 1 | 0 | 6 | 50 |
| Control | male | inguinal hernia repair | IIB | 17 | 15 | 92 | 64 | 3 | 6 | 0 | 3 | 1 | 10 | 45 |
| HT7 | female | inguinal hernia repair | IIB | 18.2 | 20 | 41 | 15 | 1 | 0 | 0 | 1 | 0 | 6 | 55 |
| HT7 | male | orchiopexy | caudal | 11.2 | 20 | 113 | 67 | 2 | 18 | 1 | 4 | 1 | 10 | 51 |
| HT7 | male | orchiopexy | caudal | 19 | 20 | 155 | 106 | 2 | 0 | 0 | 1 | 0 | 7 | 47 |
| HT7 | male | inguinal hernia repair | IIB | 13 | 15 | 61 | 43 | 4 | 16 | 1 | 4 | 1 | 11 | 25 |
| Control | female | umbilical hernia repair | RSB | 11 | 10 | 59 | 27 | 3 | 1 | 0 | 1 | 0 | 7 | 48 |
| Control | male | inguinal hernia repair | IIB | 22.3 | 25 | 56 | 18 | 1 | 15 | 1 | 1 | 0 | 5 | 38 |
| Control | male | hydrocelectomy | caudal | 18.5 | 20 | 151 | 103 | 1 | 0 | 0 | 1 | 0 | 5 | 52 |
| Control | male | inguinal hernia repair | IIB | 22 | 30 | 38 | 15 | 2 | 9 | 0 | 1 | 0 | 5 | 69 |
| HT7 | female | inguinal hernia repair | IIB | 13.5 | 45 | 87 | 64 | 2 | 5 | 0 | 1 | 0 | 5 | 38 |
| HT7 | female | inguinal hernia repair | IIB | 22.8 | 25 | 42 | 14 | 2 | 0 | 0 | 1 | 0 | 4 | 33 |
| HT7 | male | inguinal hernia repair | IIB | 12 | 15 | 46 | 20 | 3 | 8 | 0 | 2 | 0 | 6 | 26 |
| HT7 | male | orchiopexy | caudal | 9.2 | 30 | 98 | 62 | 1 | 12 | 1 | 1 | 0 | 5 | 44 |
| HT7 | male | orchiopexy | caudal | 13 | 15 | 132 | 82 | 3 | 0 | 0 | 1 | 0 | 5 | 34 |
| Control | female | inguinal hernia repair | IIB | 19 | 40 | 30 | 9 | 1 | 0 | 0 | 1 | 0 | 5 | 40 |
