# Supplementary material for: Unilateral electrical stimulation of the heart 7 acupuncture point to prevent emergence agitation in children: A prospective, double-blinded, randomized clinical trial
Source: PLoS One. 2018 Oct 10;13(10):e0204533. doi: 10.1371/journal.pone.0204533 (PMC6179240; doi:10.1371/journal.pone.0204533)
Supplement: S2 File — (DOCX) [file pone.0204533.s004.docx]

| **Medical reserch plan** |  | |
| --- | --- | --- |
|  |  | |
| Ⅰ　Summery of study |  | |
| 1. Summery of study | The effect of electrical stimuration using neuromuscular transmission monitor at unilateral HT7 acupoint on preventing emergence agitation in children. | |
| 2. Name of lead principal investigator  Division name | Nobuhito Nakamura  Department of Anesthesiology | |
| 3. Other investigator | Takahiro Mihara | |
| 4. Condition | Inclusion criteria：Children (ages 18–96 months) who were scheduled  to undergo day procedure of otolaryngology, urology, ophthalmology  or surgery under general anaesthesia.  Exclusion criteria：developmental delay, usage of sedatives. | |
| 5. Policy of selecting subject | none | |
| 6. Objective of this study | Emergence agitation(EA) is a major postoperative problem in pediatric anesthesia. Incidence of EA is more than 50% according to some study. Prevention of EA is important because EA may lead to self-injury , dislodgement their catheter or stress to caregivers.  Previous studies have reported that EA was effectively prevented by stimulating the heart 7 (HT7) acupuncture point using a capsicum plaster or needle. But, it is difficult to perform acustimulation using needle for many anestheologist who are not familiar to acupuncture.  Our group revealed that stimulating bilateral HT7 acupuncture point using a neuromuscular transmission monitor decreased EA incidence.  To easily utilize this method in a clinical situation, we need to optimize the electrical stimulation conditions of HT7. Bilateral stimulation is a barrier to clinical use because two machines are needed for one patient.  The objective of this study was to examine the efficacy of unilateral electrical stimulation of HT 7 using a neuromuscular transmission monitor to prevent EA in paediatric patients. | |
| 7. History of the ethics committee application | none | |
| 8. Method of study | Unilateral right-side stimulation of the HT 7 acupuncture point was performed on patients using a single-twitch electrical stimulus throughout the surgery.  Randomization: sequentially numbered envelope with a computer-generated random allocation.  Trained observers, who were blinded to the patient allocations, assessed and recorded the patient’s recovery condition, including the PAED score in the PACU. | |
| 9. Period of study | Approximately 6 months | |
| 10. Facilities where study take place | Operating room | |
| 11. Cost burden of the subject | none | |
| 12. Presence or absence of insurance adaptation | No expense is incurred by using the neuromuscular transmission monitor. | |
| 13. Expected result and disclosure of result | It is predicted that postoperative delirium will decrease in HT7-stimulated patients. The results will be disclosed. | |
| 14. Predicted risks, disadvantages and burdens | Although the risk in this study is expected to be low, redness at the stimulation site, motor paralysis, etc. may also occur. | |
| 15. Type and amounts of samples, Method of collecting samples | No sample collection scheduled | |
| 16. Target sample size | 100 patients (determined by power analysis) | |
| 17. Acquisition of personal information | ■①Yes　　　　　　　　　　　　　　　　　　　　　　　　　　　　　　□②No | |
| 18. In case of ① in 17, methods of personal information protection | ■①Unlinkable anonymizing　　　　　　　　　　　　　　　　　　　　　　　　　　　　　□②Linkabke anonymizing | |
| 19. In case of ② in 18, methods of linkable anonymizing |  | |
|  |  | |
| Ⅱ　Collaborating research institutes |  | |
| 1. Presence of a collaborating reseach institutes | □①Yes　　　　　　　　　　　　　　　　　　　　　　　　　　　　　　　　　　　　　■②No | |
| 2. Name and address of a collaborating reseach institutes | None | |
| 3. Approval from the ethics committee of the joint research institute. | □①Yes　　　　　　　　　　　　　　　　　　　　　　　　　　　　　　　　　　□②No | |
| 4. In case of ② in 3, Schedule of deliberation at the ethics committee of the joint research institute. |  | |
| 5. Roles of collaborating research institutes | □①sample collection　　　　　　　　　　　　　　　　　　　　　　　　　　　　　　　□②analysis of sample　　　　　　　　　　　　　　　　　　　　　　　　　　　　　　　　　　　　　　　□③other（　　　　　　　　　　　　　　　　） | |
| 6. In case of ① in 5, Institute where to anonymize. | □①Our center.　　　　　　　　　　　　　　　　　　　　　　　　　　　　　　　　　　　　□②Other institute. | |
| 7. Whether or not samples collected at our center are sent to joint research institutes | □①Yes　　　　　　　　　　　　　　　　　　　　　　　　　　　　　　　　　　　　　□②No | |
| 8. In case of ① in 7, Institute where to anonymize. | □①Our center.　　　　　　　　　　　　　　　　　　　　　　　　　　　　　　　　　　　　　　　　　　　　　　　　　　　　　　　　　　　　　　　　　　　　　□②Other（　　　　　　　　　　　　　　　　） | |
|  |  | |
| Ⅲ　Informed consent |  | |
| 1. Those who explain to obtain consent | Anesthesiologists participating in the study | |
| 2. Explanatory document / consent form for participant | ■①Described in separate sheet　　　　　　　　　　　　　　　　　　　　　　　　　　　　　　　　　　□②Other format（　　　　　　　　　　　　　） | |
| 3. Presence of explanatory documents for children | □①Yes　　　　　　　　　　　　　　　　　　　　　　　　　　　　　　　　　　■②No | |
| 4. In case of ② in 3, reason for absence of explanatory documents for children. | Explanations about emegence agitation are expected to be difficult for children of the target age to understand． | |
| 5. Items described in the explanation document | ①Affiliation, title, name of researcher　　　　　　　　　　　　　　　　　　　　　　　　　　　　　　　　　　　　　　　　　　　　　　　　　　　　　　　②Significance, objective and method of research　　　　　　　　　　　　　　　　　　　　　　　③About disclosure of research plan　　　　　　　　　　　　　　　　　　　　　　　　　　　　　　　　　　　　　　　　　　　　　④Study participation is optional　　　　　　　　　　　　　　　　　　　　　　　　　　　　　　　　⑤Do not receive disadvantages without acceptance　　　　　　　　　　　　　　　　　　　　　　⑥Collection, storage, use and disposal of samples  ⑦Privacy is protected when reporting research results  ⑧Attribution of intellectual property rights arising from research  ⑨Possibility of research results for future treatment  ⑩Research period　　　　　　　　　　　　　　　　　　　　　　　　　　　　　　　　　　　　⑪Withdrawal of consent is possible at any time　　　　　　　　　　　　　　　　　　　　　⑫Costs related to research　　　　　　　　　　　　　　　　　　　　　　　　　　　　⑬Compensation associated with research　　　　　　　　　　　　　　　　　　　　　　　　　　　　⑭Contacts such as complaints　　　　　　　　　　　　　　　　　　　　　　　　　　　　　　　　⑮Profits, disadvantages, burdens, and expected results brought by providers of samples etc.　　　　　　　　　　　　　　　　　　　　　　　　　　　　　　　　　⑯Method of protection of personal information　　　　　　　　　　　　　　　　　　　　⑰Possibility to provide personal information to other agencies　　　　　　　　　　　　　　　　　　　　　　　　　　　⑱About the use of samples etc. in case of extension of period  ⑲Explanation of the result when the research succeeded | |
|  |  | |
| Ⅳ　Legally acceptable representative |  | |
| 1. Whether the sample from persons corresponding to the following items is to be studied | □①16 years of age or older minors　　　　　　　　　　　　　　　　　　　　　　　　　　　　　　　□②Minors under 16 | |
| 2. If any one of the items in 1, is there a direct benefit to the person? | □①Yes　　　　　　　　　　　　　　　　　　　　　　　　　　　　　　　　　　　　　□②No | |
| 3. The reason for the case of ① in 2 |  | |
| 4. Perspective regarding to legally acceptable representative |  | |
| Ⅴ　Sample provision to institutions other than collaborative research institutes of this research (including cases of outsourcing analysis etc.) | |  |
| 1. Will you provide samples etc. to other organizations? | □①Yes　　　　　　　　　　　　　　　　　　　　　　　　　　　　　　　　　　　■②No | |
| 2. In case of ① in 1, necessity of providing samples to external organizations |  | |
| 3. In case of ① in 1, destination organization name |  | |
| 4. Method of anonymization at the provider |  | |
| 5. Name of the person in charge at the destination, responsibility system, contents of the contract planned |  | |
|  |  | |
| Ⅵ　Sample preservation |  | |
| 1. Whether samples should be stored at our center during the research period | □①Yes　　　　　　　　　　　　　　　　　　　　　　　　　　　　　　　　　■②No | |
| 2. Do you store samples at our center after the research period? | □①Yes　　　　　　　　　　　　　　　　　　　　　　　　　　　　　　　　　■②No | |
| 3. The reason for the case of ① in 2. |  | |
| 4. Whether to save the sample at the collaborative research institution after the research period | □①Yes　　　　　　　　　　　　　　　　　　　　　　　　　　　　　　　　　■②No | |
| 5. The reason for the case of ① in 4. |  | |
| 6. Method for disposing samples. |  | |
|  |  | |
| Ⅶ　Procurement method of research funding |  | |
| 1. Method of procuring research funding | Procurement of research funding in this research is not necessary. | |
|  |  | |
| Ⅷ　About compensation relating to the medical research |  | |
| 1. Presence of compensation accompanying research | □①Yes  ■②No | |
| 2. In case 1 in ①, contents of compensation |  | |
|  |  | |
| Ⅸ　Intellectual property right |  | |
| 1. About Intellectual Property Rights | None | |
|  |  | |
| X　 Conference presentation of research results |  | |
| 1. Are there plans for presentations at academic conferences? | ■①Yes　　　　　　　　　　　　　　　　　　　　　　　　　　　　　　　　　　□②No | |
| 2. In case of ①in 1,points to be noted | None | |
|  |  | |
| XI　 Disclosure of research plan |  | |
| １. Is disclosure of research plan possible? | ■①Possible　　　　　　　　　　　　　　　　　　　　　　　　　　　　　　　　　　□②Partly impossible　　　　　　　　　　　　　　　　　　　　　　　　　　　　　　　　　　　　　　□③Impossible | |
| 2. In case of ②　in 1, item number not to disclose | None | |
| 3. The reason for the case of ② or ③ in 1. | None | |
