# Supplementary material for: Unilateral electrical stimulation of the heart 7 acupuncture point to prevent emergence agitation in children: A prospective, double-blinded, randomized clinical trial
Source: PLoS One. 2018 Oct 10;13(10):e0204533. doi: 10.1371/journal.pone.0204533 (PMC6179240; doi:10.1371/journal.pone.0204533)
Supplement: S3 File — (DOCX) [file pone.0204533.s005.docx]

| 医学研究計画書 |  | |
| --- | --- | --- |
|  |  | |
| Ⅰ　概要 |  | |
| 1.研究の名称 | 小児における筋弛緩モニターを用いた片側の経穴（ＨＴ７）の  刺激による麻酔後覚醒時せん妄の予防効果の検討 | |
| 2.研究責任者・所属・職名・氏名 | 中村信人・麻酔科 | |
| 3.その他の実施担当者 | 水原敬洋 | |
| 4.対象とする疾患名 | 適格基準：全身麻酔下に手術を受ける1才6か月~8才の患者．  対象手術：耳鼻科，眼科，泌尿器科，外科の日帰り手術  除外基準：精神発達遅滞，向精神薬内服中の患者、 | |
| 5.対象者を選ぶ方針 |  | |
| 6.研究の意義及び目的 | 小児において麻酔覚醒後せん妄は50％以上に起こる合併症  で，自傷や点滴ライン自己抜去のリスク，看護担当者のス  トレスが上昇するため予防が急務である．  手首にある経穴（HT7）を刺激することで覚醒後せん妄が予  防できるとの報告があるが，一般麻酔科医には実行が難し  い針治療であり普及には制限がある．当科では，すでに麻  酔科医が通常使用する筋弛緩モニターによる電気刺激を両側  前腕のHT7に行うことで覚醒時せん妄予防効果が得られること  をランダム化比較試験において確認している。  しかし、今後HT7刺激を臨床応用するに当たってはより簡便  で有効な方法を模索する必要がある。現在の両側の刺激では  筋弛緩モニターが一人につき２台必要となることから、実  際の臨床現場で多くの患者に施行するのは困難である。片  側のＨＴ７の刺激で予防効果が得られることが分かればさ  らに広く臨床応用が可能となると考えられる。  そこで本研究では，筋弛緩モニターを用いた片側のHT7の刺  激を行うことによる覚醒時せん妄の予防効果をランダム化比  較試験を行い検討する． | |
| 7.倫理委員会申請への経緯 |  | |
| 8.研究方法 | HT7の刺激方法：筋弛緩モニターを使用し、１Hz、50mAで手術開始から  終了まで刺激を継続  ランダム化：封筒法によりランダム化  評価者：患者割付を知らない回復室の看護師もしくは麻酔科医 | |
| 9.研究期間 | 約6か月間 | |
| 10.研究実施場所 | 手術室 | |
| 11.対象者の費用負担 | なし | |
| 12.保険適用の有無 | 筋弛緩モニターの使用の有無に関して費用は発生しない | |
| 13.予測される成果及び研究成果の開示 | HT7刺激患者では術後のせん妄が減ると予測される。結果は開示する。 | |
| 14.予測される危険性・不利益・負担 | 本研究における危険性は低いと予測されるが、刺激部位の発赤、  運動麻痺などが起こる可  能性もある。 | |
| 15.試料等の種類と量、その採取方法 | 試料採取予定なし | |
| 16.研究対象予定人数 | 100人（power analysisを行い決定した） | |
| 17.個人情報取得の有無 | ■①有り　　　　　　　　　　　　　　　　　　　　　　　　　　　　　　□②無し | |
| 18.17で①の場合個人情報の保護の方法 | ■①連結不可能匿名化　　　　　　　　　　　　　　　　　　　　　　　　　　　　　□②連結可能匿名化 | |
| 19.18で②の場合、その方法 |  | |
|  |  | |
| Ⅱ　共同研究機関 |  | |
| 1.共同研究機関の有無 | □①あり　　　　　　　　　　　　　　　　　　　　　　　　　　　　　　　　　　　　　■②なし | |
| 2.共同研究機関の名称・住所・研究者氏名 |  | |
| 3.共同研究機関の倫理委員会の審議を経ているか。 | □①はい（結果通知の写しを添付）　　　　　　　　　　　　　　　　　　　　　　　　　　　　　　　　　　□②いいえ | |
| 4.３で②の場合、今後審議を経る予定があるか。 |  | |
| 5.共同研究機関の役割 | □①試料の採取　　　　　　　　　　　　　　　　　　　　　　　　　　　　　　　□②試料の解析　　　　　　　　　　　　　　　　　　　　　　　　　　　　　　　　　　　　　　　□③その他（　　　　　　　　　　　　　　　　） | |
| 6.5で①の場合、どこで匿名化を行うか | □①当センターで行う　　　　　　　　　　　　　　　　　　　　　　　　　　　　　　　　　　　　□②当センターで行わない | |
| 7.当センターで採取した試料等を共同研究機関への送付の有無 | □①あり　　　　　　　　　　　　　　　　　　　　　　　　　　　　　　　　　　　　　□②なし | |
| 8.7で①の場合、どこで匿名化を行うか | □①当センターで行う　　　　　　　　　　　　　　　　　　　　　　　　　　　　　　　　　　　　　　　　　　　　　　　　　　　　　　　　　　　　　　　　　　　　　□②その他（　　　　　　　　　　　　　　　　） | |
|  |  | |
| Ⅲ　インフォームド・コンセント |  | |
| 1.同意を得るために説明を行う者 | 麻酔担当者 | |
| 2.提供者に渡す説明文書／同意書 | ■①別添　　　　　　　　　　　　　　　　　　　　　　　　　　　　　　　　　　□②その他（　　　　　　　　　　　　　） | |
| 3.こども用説明文書の有無 | □①有　　　　　　　　　　　　　　　　　　　　　　　　　　　　　　　　　　■②無 | |
| 4.3で②の場合その理由 | 覚醒時せん妄に関する説明は対象とする年齢の小児が理解  することが困難と予想されるため． | |
| 5.説明文書に記載されている事項 | ①研究者等の所属・職・氏名　　　　　　　　　　　　　　　　　　　　　　　　　　　　　　　　　　　　　　　　　　　　　　　　　　　　　　　　②研究の意義・目的・方法　　　　　　　　　　　　　　　　　　　　　　　③研究計画書の開示について　　　　　　　　　　　　　　　　　　　　　　　　　　　　　　　　　　　　　　　　　　　　　④研究への参加が任意であることについて　　　　　　　　　　　　　　　　　　　　　　　　　　　　　　　　　⑤承諾をしなくても不利益を受けないこと　　　　　　　　　　　　　　　　　　　　　　　⑥試料等の採取・保存・使用・廃棄について  ⑦研究結果の報告についてプライバシーが保護されることについて  ⑧研究から生じる知的財産権の帰属について　　　　　　　　　　　　　　　　　　　　　　　　　　　　　⑨研究成果の今後の治療等への利用可能性について  ⑩研究期間　　　　　　　　　　　　　　　　　　　　　　　　　　　　　　　　　　　　　⑪承諾の撤回は随時可能なことについて　　　　　　　　　　　　　　　　　　　　　⑫研究に係る費用について　　　　　　　　　　　　　　　　　　　　　　　　　　　　⑬研究に伴う補償について　　　　　　　　　　　　　　　　　　　　　　　　　　　　⑭苦情などの相談先について　　　　　　　　　　　　　　　　　　　　　　　　　　　　　　　　⑮試料等の提供者にもたらされる利益、不利益及び負担・予測される結  果について　　　　　　　　　　　　　　　　　　　　　　　　　　　　　　　　　　⑯個人情報の保護の方法　　　　　　　　　　　　　　　　　　　　　　　　　　⑰試料等や個人情報を他の機関に提供する可能性、その方法　　　　　　　　　　　　　　　　　　　　　　　　　　　　⑱期間延長の場合の試料等の使用について　　　　　　　　　　　　　　　　　⑲研究が成功した場合の結果の説明の希望 | |
|  |  | |
| Ⅳ　代諾 |  | |
| 1.次に該当する者からの試料を研究の対象とするのか | □①16歳以上の未成年から　　　　　　　　　　　　　　　　　　　　　　　　　　　　　　　□②16歳未満の未成年から | |
| 2.1のうちいずれかに該当する場合、本人に直接の利益があるか | □①あり　　　　　　　　　　　　　　　　　　　　　　　　　　　　　　　　　　　　　□②なし | |
| 3.2で①のときその理由 |  | |
| 4.代諾者選定に関する考え方 |  | |
| Ⅴ　本研究の共同研究機関以外の機関への提供（解析を外部委託する場合などを含む） | |  |
| 1.試料等を他機関に提供するか | □①提供する　　　　　　　　　　　　　　　　　　　　　　　　　　　　　　　　　　　■②提供しない | |
| 2.1で①の場合、その必要性 |  | |
| 3.1で①の場合、提供先機関名 |  | |
| 4.提供元における匿名化の方法 |  | |
| 5.提供先における責任者の氏名、責任体制、予定する契約の内容 |  | |
|  |  | |
| Ⅵ　試料等の保存 |  | |
| 1.研究期間中試料等を当センターで保存するか | □①保存する　　　　　　　　　　　　　　　　　　　　　　　　　　　　　　　　　■②保存しない | |
| 2.研究期間後試料等を当センターで保存するか | □①保存する　　　　　　　　　　　　　　　　　　　　　　　　　　　　　　　　　■②保存しない | |
| 3.2で①のときその理由 |  | |
| 4.研究期間  後試料等を共同研究機関で保存するか | □①保存する　　　　　　　　　　　　　　　　　　　　　　　　　　　　　　　　　■②保存しない | |
| 5.4で①のときその理由 |  | |
| 6.試料等を廃棄する際の方法 |  | |
|  |  | |
| Ⅶ　研究資金の調達方法 |  | |
| 1.研究資金の調達方法 | なし | |
|  |  | |
| Ⅷ　当該医学研究に伴う補償について |  | |
| 1.研究に伴う補償の有無 | □①有り  ■②無し | |
| 2.1で①の場合その内容 |  | |
|  |  | |
| Ⅸ　知的財産権 |  | |
| 1.知的財産権について | なし | |
|  |  | |
| X　研究結果の発表について |  | |
| 1.学会等での発表の有無 | ■①有り　　　　　　　　　　　　　　　　　　　　　　　　　　　　　　　　　　□②無し | |
| 2.1で①の場合の留意事項について |  | |
|  |  | |
| XI　計画書の開示 |  | |
| １.計画書開示の可否 | ■①可　　　　　　　　　　　　　　　　　　　　　　　　　　　　　　　　　　□②部分的に不可　　　　　　　　　　　　　　　　　　　　　　　　　　　　　　　　　　　　　　□③不可 | |
| 2.1で②のとき開示を望まない項目番号 | □①可　　　　　　　　　　　　　　　　　　　　　　　　　　　　　　　　　　□②不可 | |
| 3.1で②または③のときその理由 |  | |
